# Supplementary material for: Cortical Layer Markers Expression and Increased Synaptic Density in Interstitial Neurons of the White Matter from Drug-Resistant Epilepsy Patients
Source: Brain Sci. 2023 Apr 6;13(4):626. doi: 10.3390/brainsci13040626 (PMC10137301; doi:10.3390/brainsci13040626)
Supplement: Supplementary file 1 [file brainsci-13-00626-s001.zip › brainsci-2304427-supplementary.pdf]

Supplementary material

Fig. S1

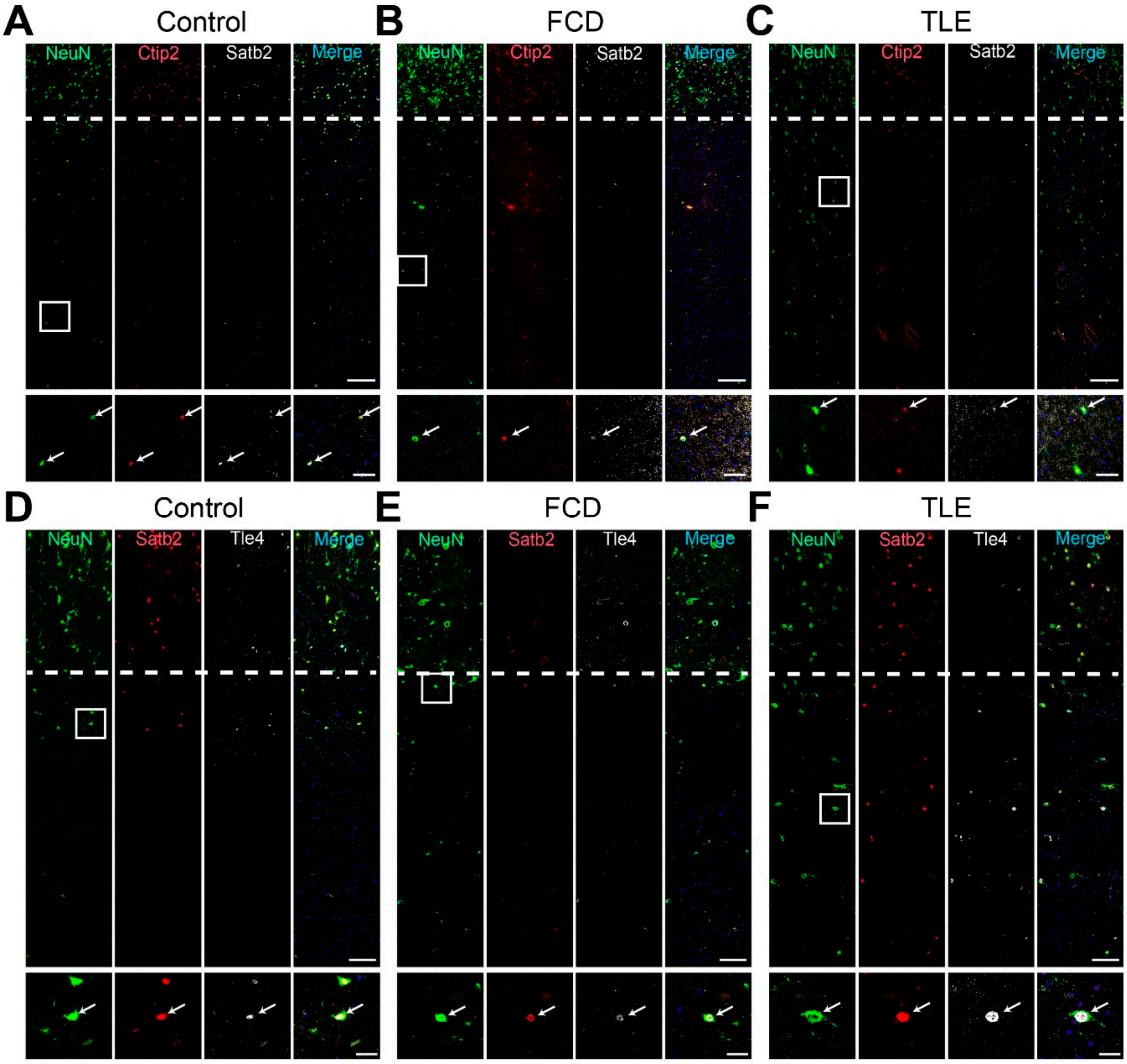

Figure. S1. Expression of cortical layer markers in interstitial neurons in the white matter of brain tissues from control, FCD and TLE.

(A) Tile scan images showing the expression of Ctip2 and Satb2 in interstitial neurons in the white matter of additional brain tissues from control, FCD **(B)** and TLE **(C)**. NeuN, neuronal marker; Satb2, upper layer cortical pyramidal neuron marker; Ctip2, deep layer cortical pyramidal neuron marker. Arrows indicated co-expression of cortical layer markers with NeuN. Scale bar, 200 μm in tile scan image (up) and 50 μm in zoom-in image (bottom). Dashed line indicated border of gray matter and white matter.

(D) Tile scan images showing the distribution of interstitial neurons expressing Satb2 and Tle4 in the white matter of brain tissues from control, FCD **(E)** and TLE **(F)**, respectively. Tle4, deep layer cortical pyramidal neuron marker. Arrows indicated co-expression of cortical layer markers with NeuN. Scale bar, 100 μm in tile scan image (up) and 20 μm in zoom-in image (bottom). Dashed line indicated border of gray matter and white matter.

Fig. S2

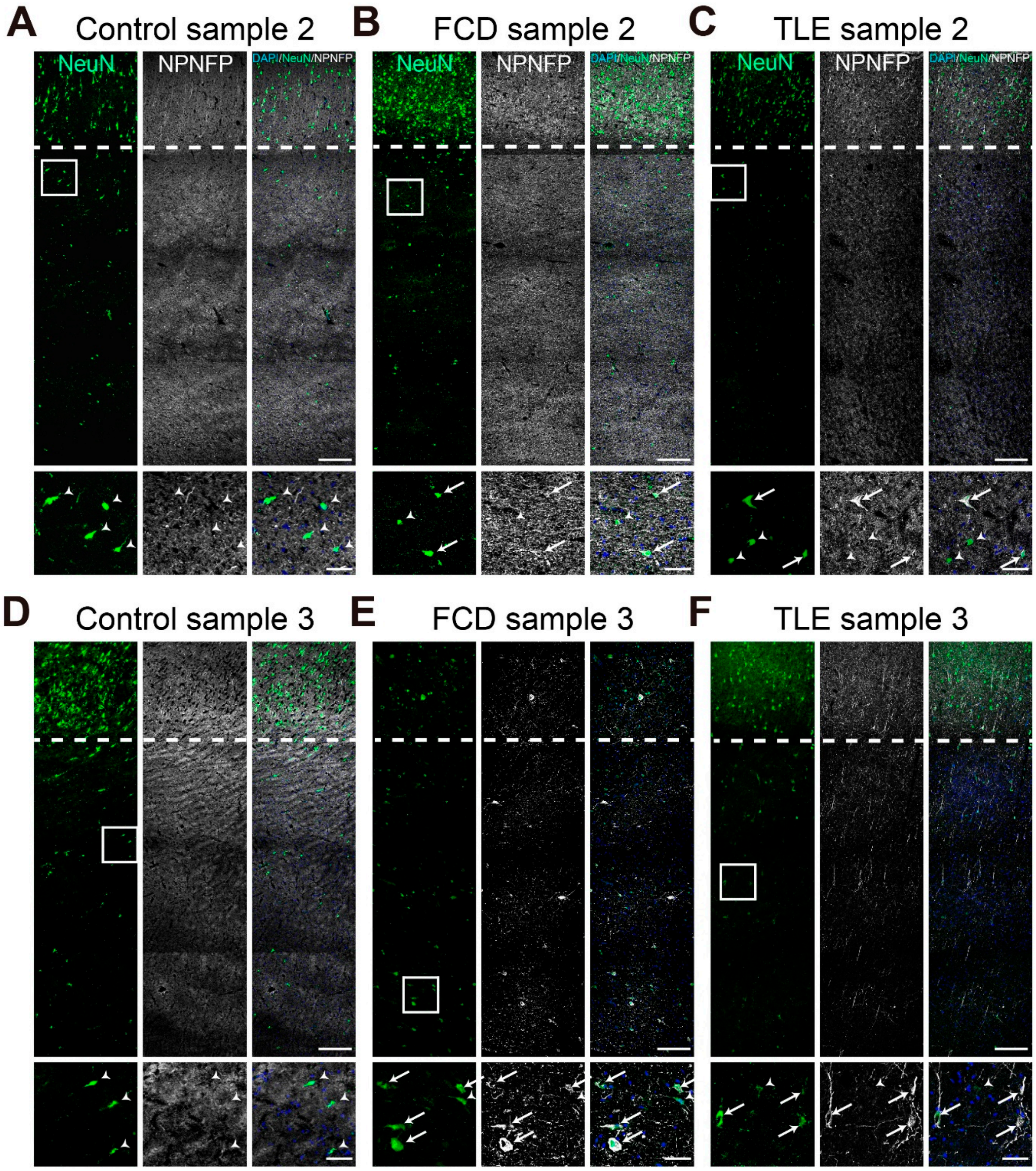

Figure. S2. Distribution of NPNFP positive interstitial neurons in the white matter of drug-resistant epilepsy patients and control brain tissue.

(A-F) Representative images from two independent brain samples (upper and lower panels) showing the expression of NPNFP in the white matter of brain tissue sections from control **(A,D)**, FCD **(B,E)** and TLE **(C,F)**. Arrows indicated co-expression of NPNFP with NeuN. Arrows indicated co-expression of NPNFP with NeuN. Arrowheads indicated no NPNFP expression were detected in interstitial neurons. Scale bar, 200 μm in the original image (up) and 50 μm in zoom-in image (bottom). Dashed line indicated border of gray matter and white matter.

Fig. S3

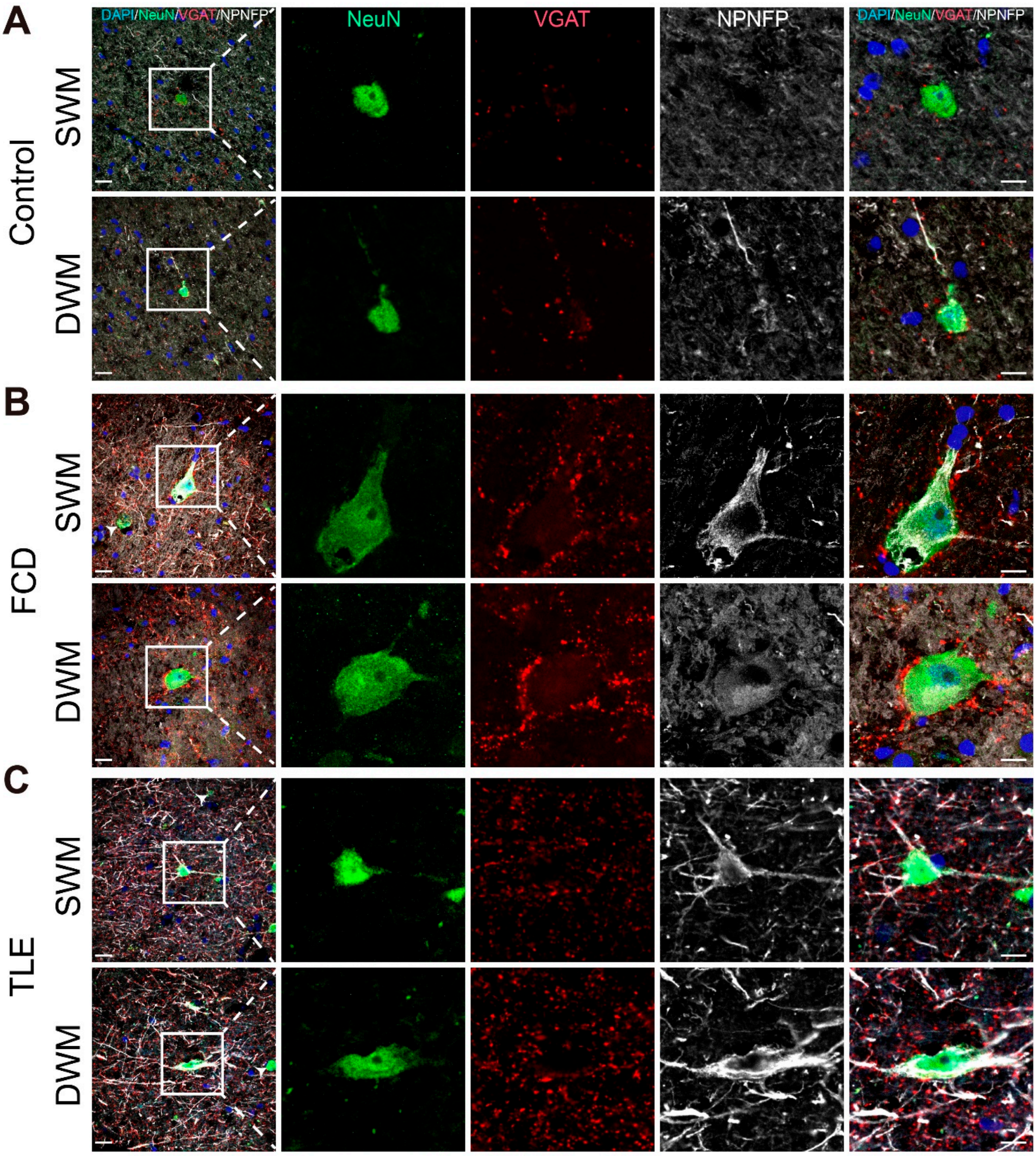

Figure. S3. Representative images showing the distribution of GABAergic synapses in the white matter of drug-resistant epilepsy patients and control brain tissue.

(A) Example images showing the distribution of GABAergic synaptic marker VGAT in the peri-soma and dendrites of interstitial neurons in the superficial and deep white matter of brain tissue sections from additional brain samples from control, FCD **(B)** and TLE **(C)**. Scale bar, 20 μm in the original image (left) and 10 μm in zoom-in image (right). SWM: superficial white matter; DWM: deep white matter.

Fig. S4

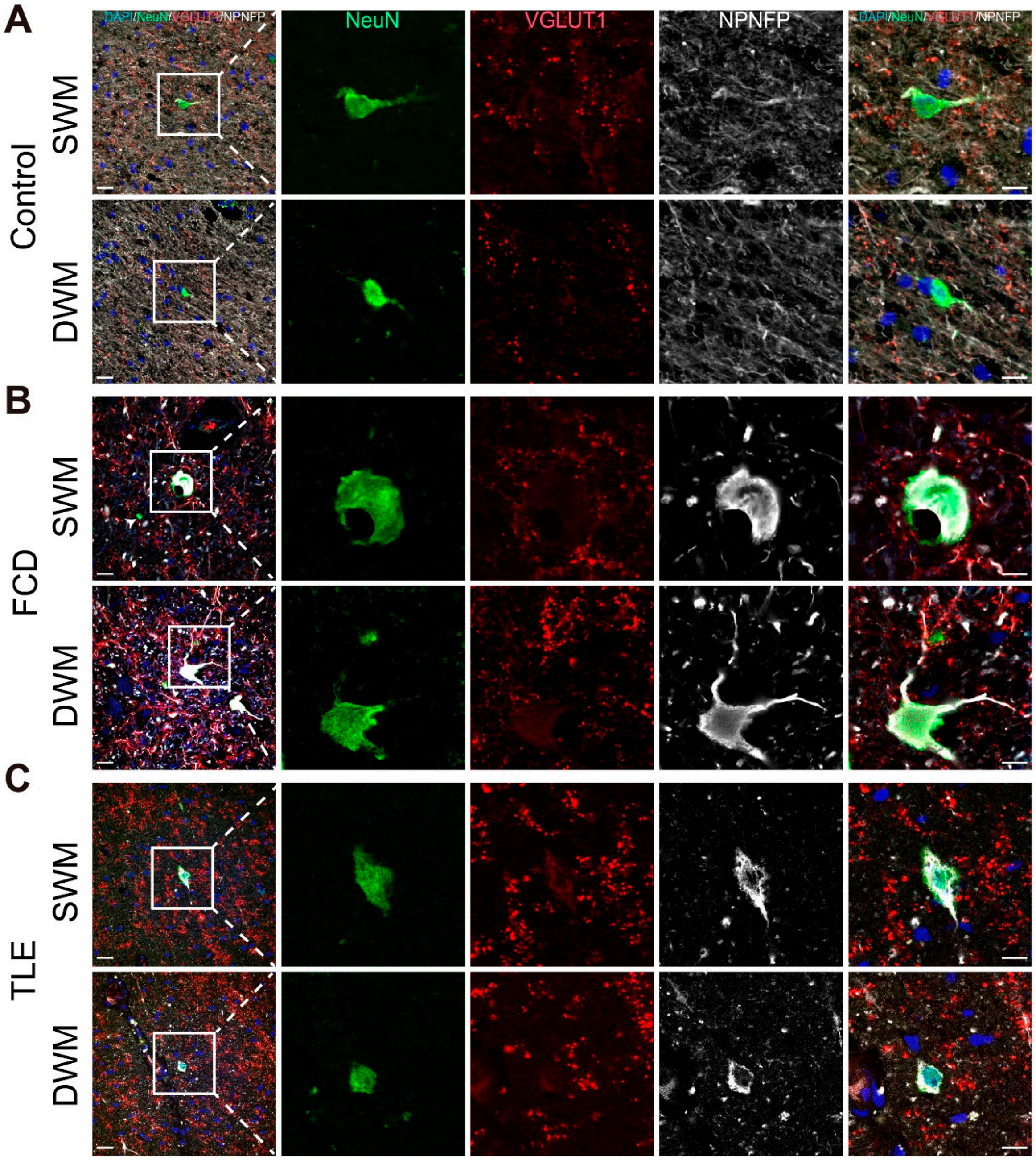

Figure. S4. Representative images showing the distribution of glutamatergic synapses in the white matter of drug-resistant epilepsy patients and control brain tissue.

(A) Example images showing the distribution of glutamatergic synaptic marker VGLUT1 in the peri-soma and dendrites of interstitial neurons in the superficial and deep white matter of brain tissue sections from additional brain samples from control, FCD **(B)** and TLE **(C)**. Scale bar, 20 μm in the original image (left) and 10 μm in zoom-in image (right). SWM: superficial white matter; DWM: deep white matter.
